# Oncovascular surgery

**DOI:** 10.1590/1806-9282.2024S103

**Published:** 2024-06-07

**Authors:** Francisco João Sahagoff de Deus Vieira Gomes, Adriana Rodrigues Vasconcelos, Ivan Vasconcelos Gomes Sahagoff, Julio Cesar Peclat de Oliveira

**Affiliations:** 1 Universidade do Estado do Rio de Janeiro, Faculty of Medical Sciences, Department of Surgery, Military Police of the State of Rio de Janeiro, Brazilian Society of Angiology and Vascular Surgery – Rio de Janeiro (RJ), Brazil.; 2 Miguel Couto Municipal Hospital, Brazilian Society of Angiology and Vascular Surgery – Rio de Janeiro (RJ), Brazil.; 3 Universidade Federal do Rio de Janeiro – Rio de Janeiro (RJ), Brazil.; 4 Brazilian Society of Angiology and Vascular Surgery, Technical Chamber of Vascular Surgery of the Regional Council of Medicine of the State of Rio de Janeiro, Vascular Surgery Technical Chamber of the Federal Council of Medicine – São Paulo (SP), Brazil.

## INTRODUCTION

Surgical intervention is the main therapeutic method for cancer cure. Aggressive resection of malignant disease, including when vascular structures are involved, has really changed the patients’ quality of life (QoL). In the past, the involvement of arteries and veins was a barrier to surgery^1,2^. Until today, many surgeons are still reluctant to operate on tumors with significant vessel involvement due to the increased inherent complexity^1,2^ due to the high complexity of those operations and the uncertainty about the long-term oncological benefit. However, in the last decade, many papers suggest that survival depends on the complete elimination of the primary pathology and tumor biology, and, therefore, it has been proven that the en bloc resection technique with vascular reconstruction shows good results, which is supported by evidence in many studies in patients with multiorgan neoplastic involvement, including pancreatic, retroperitoneal, renal, and limb tumors^2^. Although many oncological surgeons are accustomed to the interventions and techniques necessary for vascular repair and also for vessel reconstruction, vascular surgeons have more practice and a bigger set of important skills that can facilitate complex oncological resolution and even reduce operative time. Careful preoperative planning by a multidisciplinary team consisting of an oncological surgeon and a vascular surgeon is essential^1,3^.

## CONCEPT

The term "oncoplastic surgery," "oncoplasty," or even "onco-reconstructive surgery" refers to the association of plastic surgery techniques with breast reconstruction simultaneously with the surgical treatment of breast cancer resection, and it has already been established in the medical world for a few decades^1^. The basic concept was to treat cancer and preserve aesthetics without compromising oncological efficacy. The feasibility of breast reconstruction brought comfort and self-esteem to those patients who were already depressed and distressed by the challenge of neoplasia, which is the main cause of death in women around the world, and this has a great impact on several pillars of their lives (psychological, sexual, affective, and social)^4^, therefore ensuring, above all, QoL. For the success of oncoplastic surgery, multidisciplinary therapy is essential.

The vascular surgeon is dedicated to treat arterial and venous diseases using drug therapy, open surgery, endovascular surgery, or hybrid techniques, and, consequently, oncological surgery is not their main focus^1^.

Oncovascular surgery (OVS) is a term similar to oncoplastic surgery that implies oncological surgery with simultaneous vascular reconstruction. OVS can be defined as surgical resection of the malignant disease with concomitant ligation or reconstruction of a large vascular structure. Experts have pointed out the increasing relevance of OVS, which includes training for surgeons who act as strategists involved in all phases of treatment: planning, execution, and post-operative follow-up^5^. The use of the term "OVS" may increase awareness of the important role of vascular surgeons in complex cancer surgeries among the public and the medical community^1^.

The concept of OVS has become increasingly popular and is already considered a determining factor for quality and safety in R0 resections aimed at curing advanced cancers^6,7^.

Ghosh et al., after reviewing several health electronic databases, reported that the published results about different neoplasms suggested that survival depended on the complete extirpation of the primary disease and the tumor’s biology, instead of vessel-related complications, and concluded that a bigger vessel involvement by a tumor mass shall not be necessarily considered a barrier against block resection and, by extension, against curative surgery. The radical surgical resection may offer the only cure or palliation chance to these patients^7^.

The vascular surgeon must act on all treatment phases: on preoperative planning, choosing the best technique and surgical access, or performing embolizations in order to decrease the circulatory contribution to the mass and reduce the bleeding during the resection; per-operative, performing resections and vessel reconstructions, avoiding and/or controlling important or fatal hemorrhage; and post-operative, diagnosing ischemic complications, venous thromboembolism (VTE), or lymphedema by lymphatic interruption or by radical ganglionar emptying.

## THE VASCULAR SURGEON’S CURRENT ROLE IN ONCOLOGICAL SURGERY

The vascular surgeon’s performance may be classified into three distinct categories: as the main surgeon of vessel-originated tumors, as a rescue surgeon against complications during the cancer surgery, and as a multidisciplinary team’s consulting surgeon in cancer treatment^1^.

### Primary surgeon for vessel-origin tumors

Vascular surgeons must treat some rare primary malignant tumors of blood vessels, such as angiosarcoma, leiomyosarcoma, and retroperitoneal sarcoma, as well as intravenous leiomyomatosis (IVL), a rare benign tumor.

### Angiosarcoma

Malignant tumors of the aorta are classified according to their originary cell: intimal angiosarcoma, medial leiomyosarcoma, and adventitial fibrosarcoma.

Angiosarcoma, an infiltrative tumor with a high rate of local recurrence and metastasis, represents less than 2% of all soft tissue sarcomas and mainly affects adult and elderly patients^8^. Reported rates of metastatic disease at presentation range from 16 to 44%, and survival ranges from 6 months to 16 months^8^. According to epidemiological research, angiosarcoma has a similar distribution between genders and can occur at any age^8^.

Thromboembolic complications are the typical clinical presentation. Several authors recommend resection of the tumor-bearing aortic region^9^, but it is not clear if this approach is beneficial to the patient. Chemotherapy and radiotherapy have been shown to be of less value for patient survival but may play a role in certain circumstances, such as inoperable tumors or metastatic complications^9^.

These tumors are difficult to diagnose preoperatively and, in most cases, are diagnosed late, resulting in a worse prognosis. Clinical suspicion of a primary angiosarcoma of the aorta, especially in cases of atypical, rapidly growing abdominal aortic aneurysm with a thrombotic mass, is essential for early diagnosis and adequate surgical management.

### Leiomyosarcoma of the vena cava

Of all types of leiomyosarcoma, vascular ones account for 2%, affecting veins five times more often^10^. Primary tumors of the inferior vena cava (IVC) are rare, and 95% of them correspond to leiomyosarcomas that present insidious, non-specific symptoms. Generally, the diagnosis is made late and, consequently, the prognosis is often poor.

Curative treatment requires aggressive surgical excision of the tumor and the involved IVC segment with clear margins. This radical approach associated with the absence of metastases can provide long-term survival and, eventually, even cure^11-13^. However, radiotherapy or chemotherapy is ineffective^1,14^.

**Figure 1:**
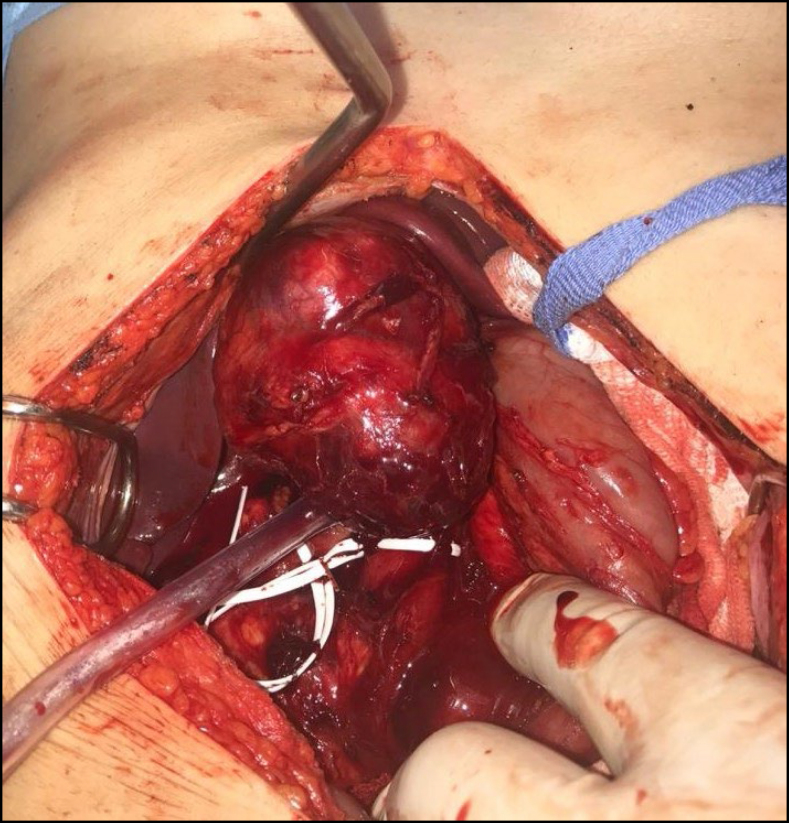
Caval leiomyosarcoma.

**Figure 2:**
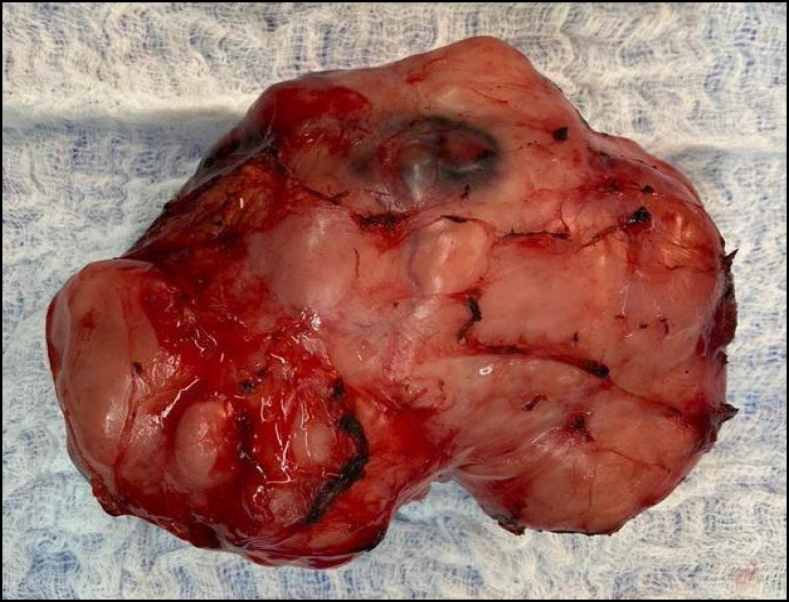
Operative piece leiomyosarcoma of the cava.

Inferior vena cava reconstruction after tumor resection can be performed in three ways: ligation without reconstruction, selective reconstruction, or routine reconstruction (Figures 1 and 2).

Choosing non-reconstruction can reduce operative time, prevent pulmonary thromboembolism from VTE of the lower limbs, avoid graft complications such as infections, and reduce the risk of high-output heart failure and the need for perennial anticoagulation.

Reconstruction, when performed, can be by primary repair, patching, or graft interposition. If the expected narrowing of the IVC is less than 50%, repair may be preferred first; when it is greater than 50%, a patch can be used. In cases where complete resection of the IVC wall is necessary, graft interposition should be chosen, knowing, however, that this is the option with the lowest patency rate. The grafts used can be an autologous vein, peritoneum, cryopreserved graft, bovine pericardium, or synthetic Dacron or poly-tetra-flour-ethylene (PTFE) prosthesis. The series, which is still small, does not allow us to establish which is the best synthetic graft or which size is best.

Preservation of renal and hepatic vein flow is particularly relevant; these veins can be resected and reimplanted. Reconstruction of the left renal vein may be unnecessary due to collateral circulation via the gonadal or adrenal gland, but reconstruction of the right renal vein is generally necessary.

### Intravenous leiomyomatosis

It is a rare tumor originated from intrauterine veins, characterized by the intravenous growth of smooth muscle nodules that are histologically benign, like vermiform projections proximally through the IVC until reaching the right chambers of the heart. The onset of IVL is in the fifth decade of life, and, as it is an extension of uterine leiomyomas, it occurs exclusively in women^15^. Patients diagnosed with this disease are often followed up in gynecology services due to having uterine leiomyoma or a previous history of hysterectomy months or even years ago^15^.

This tumor’s etiology is not completely understood, and there are two main theories: one proposes that the origin of the tumor is in the smooth muscle cells of the venous wall, where the intravenous proliferation of the tumor originates, while the other suggests that IVL appears through direct invasion of the venous system by the adjacent tumor^15^.

The clinical presentation varies greatly depending on the extent of the tumor, ranging from completely asymptomatic (in most cases) to sudden death. The complaints are of hypogastric pain or metrorrhagia^15^. As soon as vascular invasion reaches larger vessels, such as the common iliac veins and the IVC, VTE and related syndromes may occur. Renal vein thrombosis, or Budd-Chiari syndrome, has also been described^15^. About 10–30% of IVL cases affect the right atrium, causing cardiac symptoms such as palpitations, syncope, dyspnea, or chest pain^15^.

Surgical management includes excision of the uterine tumor, bilateral oophorectomy, and intravenous tumor removal, but which technique should be adopted to remove the tumor from the pelvis to the heart remains controversial. The intervention can be in one or two stages, with or without cardiopulmonary bypass, via laparotomy or laparotomy associated with median sternotomy.

Intravenous leiomyomatosis rarely embolizes during operative removal. These tumors adhere firmly to the hypogastric vein, where they originate and invade the systemic circulation, adhering to the IVC at the confluence of the ovarian vein. Tumor masses in the IVC and right atrium are generally mobile and do not adhere to the venous walls.

The characteristics of this tumor, as well as the biology of its growth, guarantee safety in performing the surgical procedure in a single abdominopelvic approach. The tumor mass must be accessed at the level of the hypogastric vein or distal IVC, followed by slight traction downward with its total removal. Per-operative echocardiography monitors the intracardiac portion of the tumor, ensuring its mobility and the absence of tumor residue at the end of the procedure.

### Retroperitoneal soft tissue sarcoma involving large vessels

Retroperitoneal soft tissue sarcomas, although rare, are tumors that are difficult to manage due to their extension and malignancy.

Treatment is based on surgical resection, ideally complete in the first approach, targeting any potential chance of cure or prolonged survival. En bloc resection of the tumor mass, adjacent organs, and tissues is the standard procedure, with vascular resection and reconstruction often required^14^.

Vascular surgeon’s work allows for a safer resection margin, with a lower risk of vascular injury and a consequent reduction in bleeding and surgical time.

### Rescue surgeon in the treatment of complications during cancer surgery

Another extremely important role of the vascular surgeon in oncological surgery is the treatment of complications of vascular origin or effect, often resulting from the surgical treatment of tumor masses.

Modern cancer treatment centers must provide vascular surgery teams among their human resources since these consultations are, in almost 60% of cases, unplanned. The most common causes of these consultations are bleeding (35%), vascular protection, limb ischemia, or vascular exposure.

**Figure 3:**
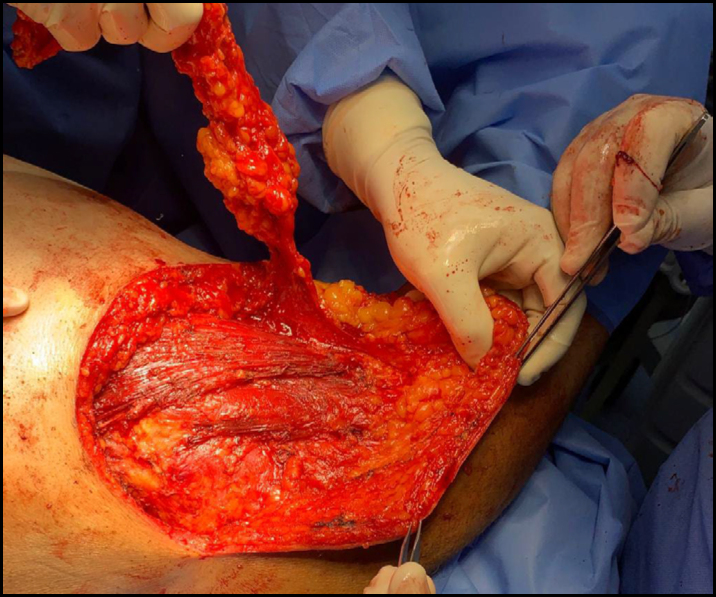
A large axillary sarcoma mobilized during dissection in the anterior region of the chest.

**Figure 4:**
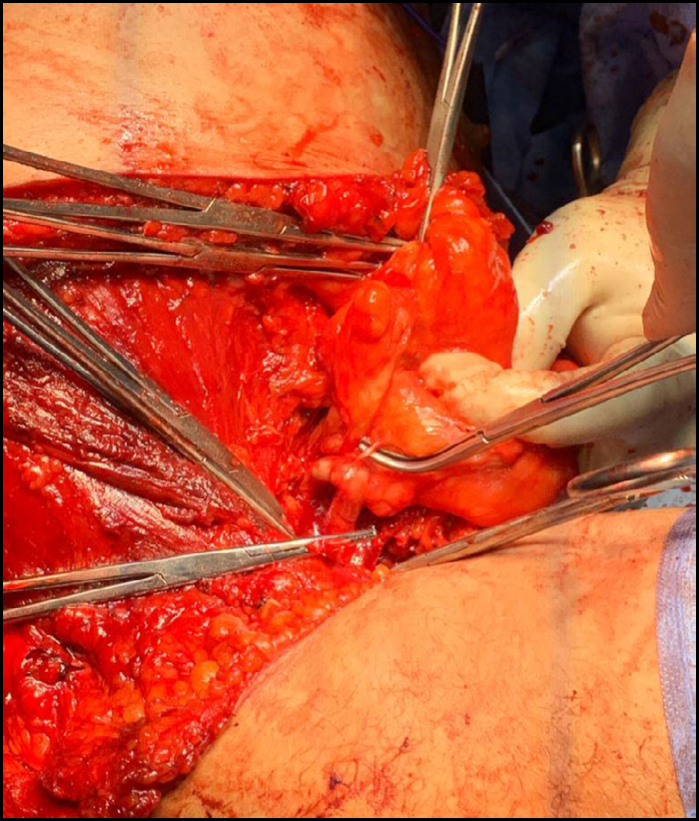
A large axillary sarcoma mobilized during dissection in the posterior region of the chest.

**Figure 5:**
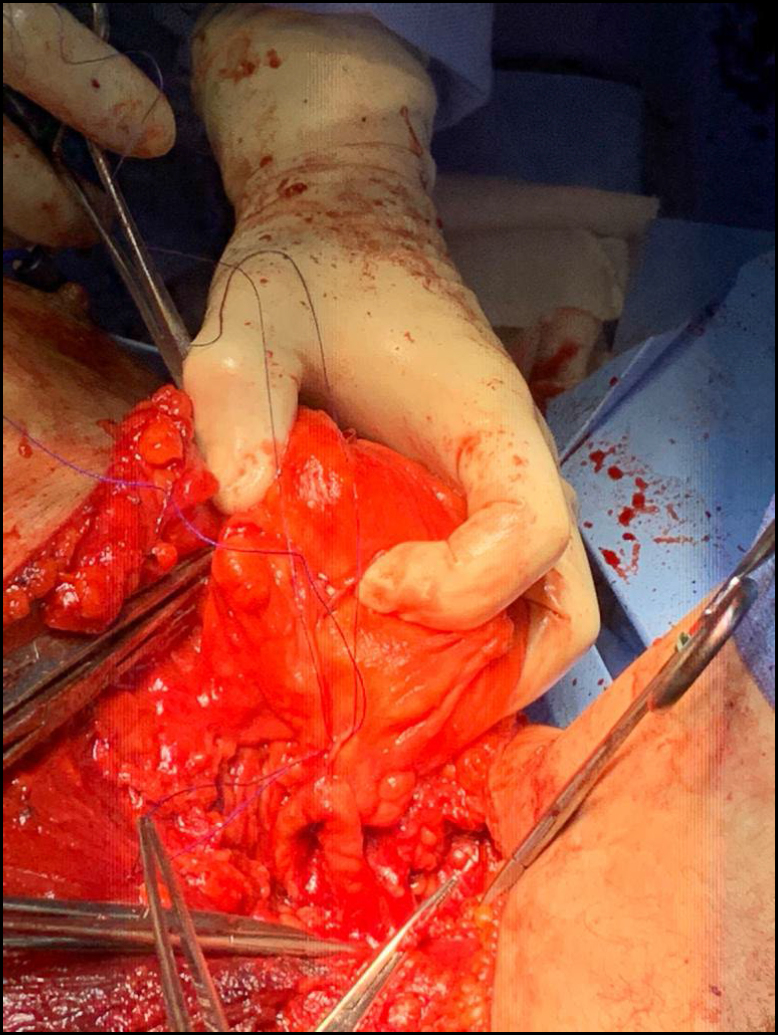
Manual mobilization and ligation of the pedicle of a large axillary sarcoma. An intimate relationship is noted between the tumor and the left axillary artery.

**Figure 6:**
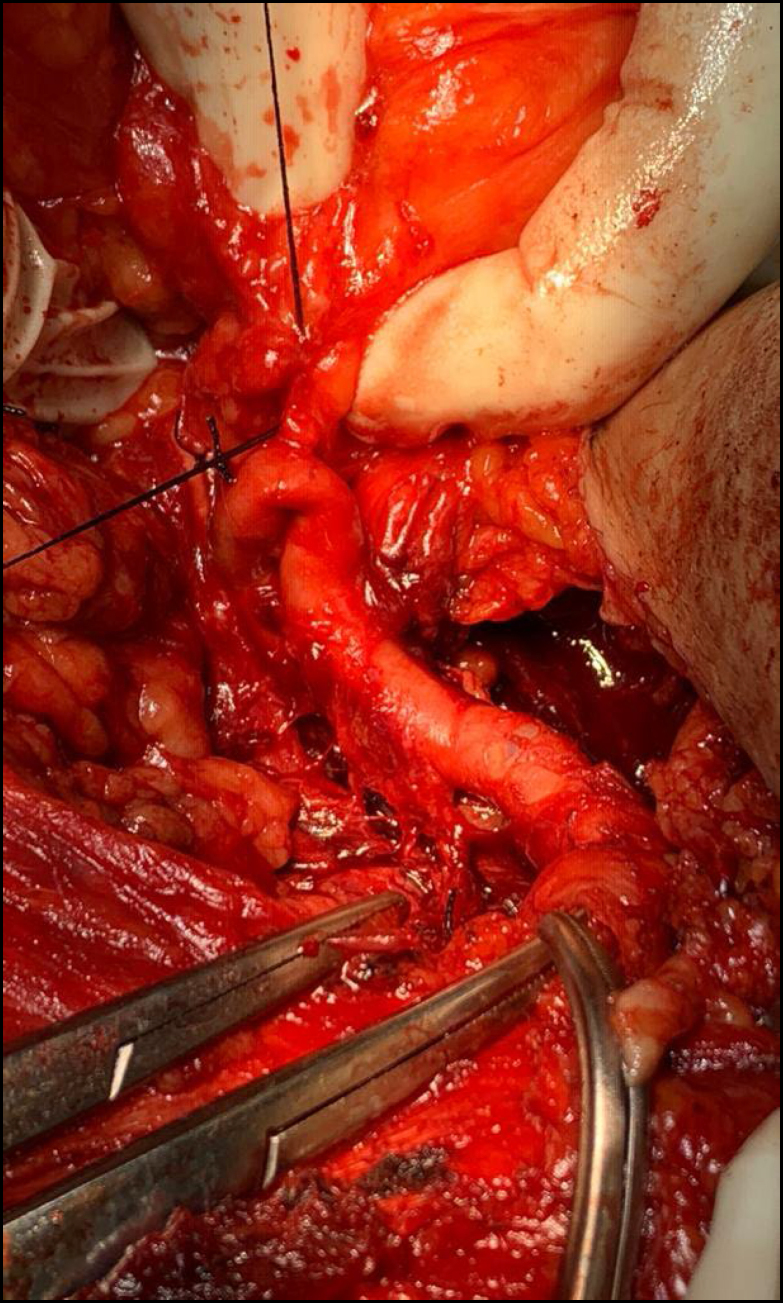
Skeletonization of the left axillary artery and ligation of one of the vascular pedicles of the large axillary sarcoma.

Among the various vascular injuries resulting from these procedures, major bleeding due to vascular laceration or transection, dissection, pseudoaneurysms, arteriovenous fistulas, acute thrombosis, and vascular contusion with late thrombosis stand out (Figures 3 to 6).

### Consultant surgeon of a multidisciplinary team in cancer surgery

The vascular surgeon must form, together with other surgeons, a cancer treatment team, especially in more advanced cases where there is involvement or invasion of large vessels.

Vascular resection, whether or not associated with reconstruction, may be necessary in the surgical treatment of a wide variety of cancers (pancreatic, invading the portal vein or hepatic artery; rectal, invading iliac vessels; thyroid tumors with invasion of the jugular vein or the carotid artery; and adrenal tumors, which involve renal vessels).

In soft tissue sarcomas of the extremities, which may involve femoral, popliteal, axillary, or brachial vessels, tumor resection can become a major challenge due to the risk of amputation, tumor recurrence, and reduced long-term survival (Figures 7–14)^16^.

**Figure 7:**
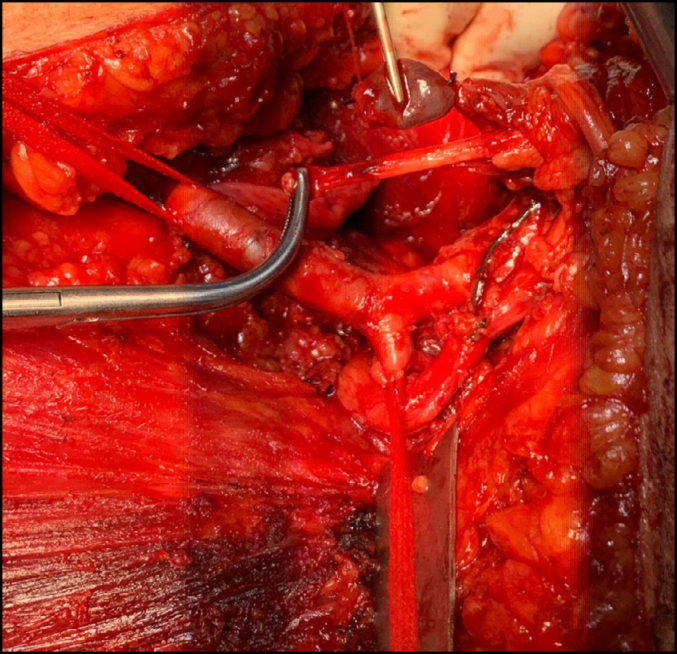
Overview of the operation after excision of the tumor mass that encompassed a branch of the brachial plexus and the axillary vein (ligated), which was too close to the axillary artery, requiring the removal of the adventitial layer, weakening the vascular wall.

**Figure 8:**
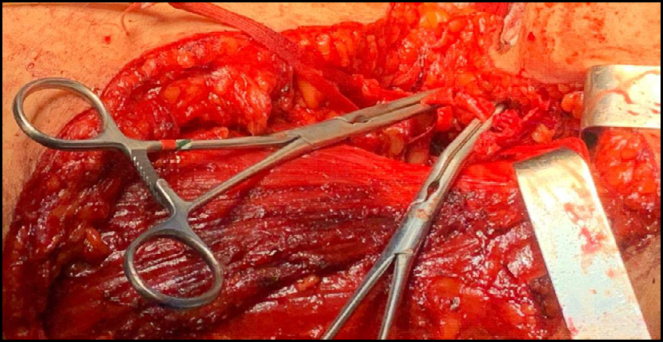
The axillary artery is clamped proximally and distally for vascular repair.

**Figure 9:**
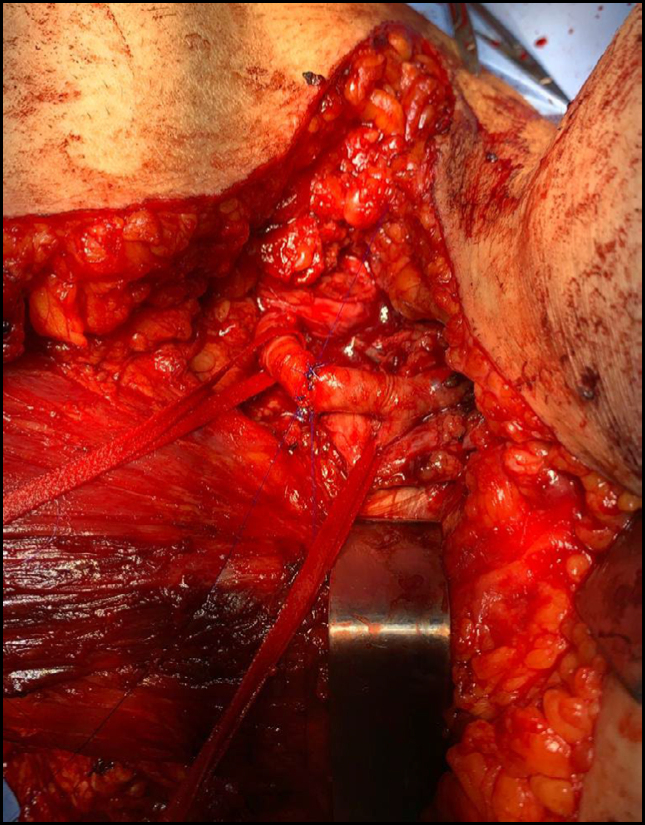
Final image after tumor resection and axillary arteriorrhaphy.

**Figure 10:**
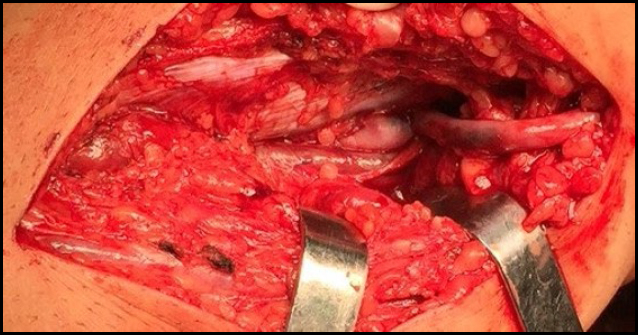
Surgical aspect of the distal anastomosis of the supra-popliteal-infrapatellar popliteal bypass before resection of popliteal synoviosarcoma.

**Figure 11:**
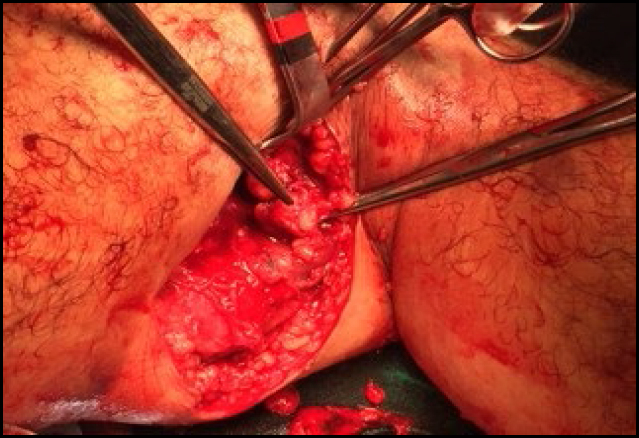
Posterior access to a popliteal synoviosarcoma.

**Figure 12:**
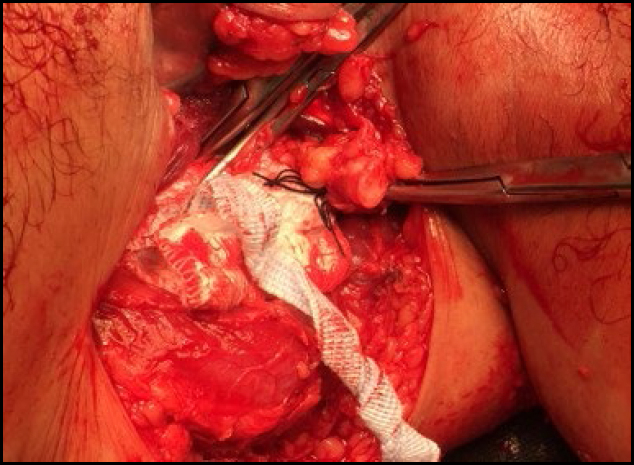
Dissection of a popliteal synoviosarcoma and ligation of the proximal and distal parts of the right popliteal artery.

**Figure 13:**
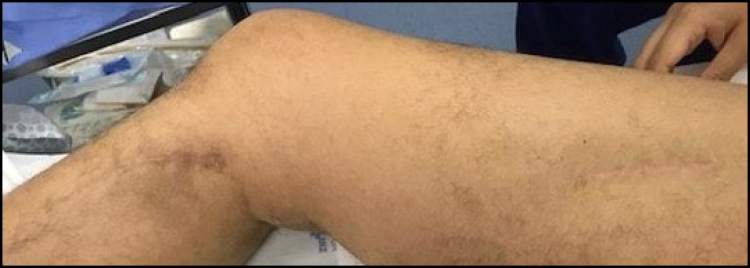
A photograph shows the scars of the medial accesses on the right leg for the suprapatellar popliteal bypass with an inverted ipsilateral saphenous vein.

**Figure 14:**
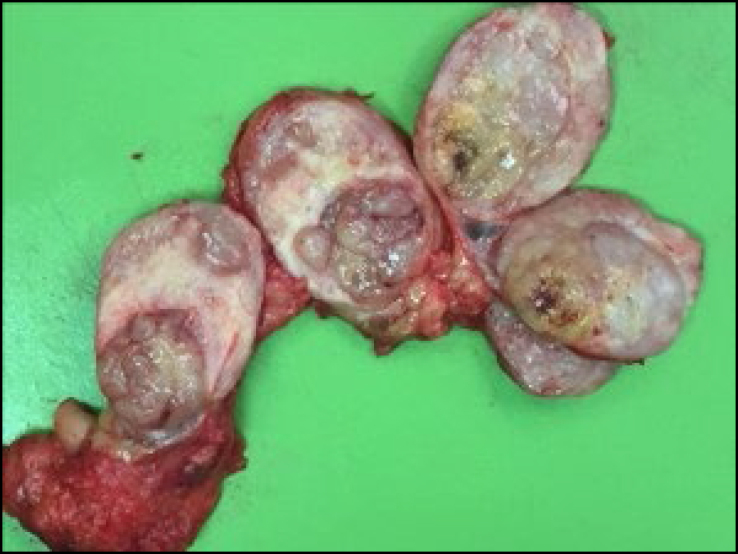
Synoviosarcoma.

## CONCLUSION

Surgical collaboration in oncological cases is technically and professionally challenging and should be an important part of the training of new surgeons, as it encourages strong interdisciplinary relationships.

Vascular surgeons must act as co-protagonists in the practice of modern oncological surgery, allowing macroscopic resection of a tumor even in the presence of invasion of large vessels.

Currently, OVS is more than a concept, which is a new vision of the multidisciplinary management of cancer patients, highly recommended for therapeutic planning, aiming for better results for these patients.
